# Systemic immunosuppression with mycophenolate mofetil to prevent corneal graft rejection after high-risk penetrating keratoplasty: a 2-year follow-up study

**DOI:** 10.1007/s00417-015-3200-2

**Published:** 2015-11-09

**Authors:** Jacek P. Szaflik, Joanna Major, Justyna Izdebska, Mieczysław Lao, Jerzy Szaflik

**Affiliations:** Department of Ophthalmology, Medical University of Warsaw, Warszawa, Poland; Independent Public University Eye Hospital, ul. J. Sierakowskiego 13, 03-709 Warszawa, Poland

**Keywords:** High-risk penetrating keratoplasty, Corneal graft failure, Corneal graft rejection, Immunosuppression, Mycophenolate mofetil

## Abstract

**Purpose:**

In this study, we aimed to evaluate the efficacy and safety of systemic immunosuppression with mycophenolate mofetil (MMF) to prevent corneal graft rejection after high-risk penetrating keratoplasty.

**Methods:**

One hundred and ninety-six consecutive patients who underwent high-risk penetrating keratoplasty defined as the presence of deep vascularization in more than two quadrants, keratouveitis, emergency keratoplasties, and retransplantations were enrolled in the study. Ninety-eight prospectively followed up patients were treated with MMF [with dose adjustment based on mycophenolic acid (MPA) serum concentration], and 98 patients were in the non–MMF-treated retrospectively assessed control group.

**Results:**

During a mean of 24 months of observation, immune reactions occurred in eight cases (8 %) and graft rejection with subsequent graft failure occurred in three cases (3 %) in the MMF group. In the control group, graft rejection occurred in 76 cases (78 %) and failure due to graft rejection occurred in 30 cases (31 %). Kaplan–Meier analysis demonstrated that 93 % of the grafts in the MMF-treated group and 47 % in the control group showed no immune rejection (*p* < 0.01, log-rank test) after a year. Cox regression analysis proved that MMF treatment decreased the risk of graft rejection 11 times (RR = 11, 95.0 % CI 4.8–25, *p* < 0.0001). Among 98 MMF-treated patients, 13 had gastric discomfort, three developed leucopenia, and two had anemia that resolved after MMF dose reduction.

**Conclusions:**

MMF treatment after high risk penetrating keratoplasty is safe and reduces the incidence of immune graft rejection and graft failure. Side effects were rare and reversible in all but one case.

## Introduction

The cornea is the most frequently transplanted tissue, with the exception of blood transfusions. The overall survival rate of low-risk corneal grafts reaches 85 %–90 % in 10 years of follow-up [1–3]. However, despite the progress in medical treatment and microsurgical procedures, penetrating keratoplasty in high-risk patients (high-risk penetrating keratoplasty [HRPK]) remains a challenging procedure due to the risk of irreversible allograft rejection. Allograft rejection occurs in 40–70 % of these patients during the first year after transplantation [4–6], and immune graft rejection is the main cause of corneal graft failure and loss of its transparency [7].

The use of cyclosporine A (CsA) is limited due to its toxicity and the risk of neocancerogenesis. Side effects such as nephrotoxicity, hepatotoxicity, hypertension, altered glucose metabolism, and gingival hyperplasia occur in up to 40 % of patients [8, 9], but probably the most threatening complication is the increased incidence of malignancy (cancers, lymphomas) [10, 11]. Reis et al. [12] and Birnbaum et al. [9] showed that mycophenolate mofetil (MMF) and CsA were equally effective and MMF treatment had a favorable safety profile, as the complications associated with MMF administration have mainly included gastrointestinal and hematologic problems (e.g., leucopenia, anemia), all of which are reversible after cessation of the drug’s dosage [13–15].

The aim of our study was to evaluate the efficacy and safety of systemic immunosuppression with MMF in the prevention of corneal graft rejection after HRPK with 2 years of follow-up.

## Material and methods

### Patients, treatment schedule, and follow-up

A total of 196 patients were included in the analysis. All patients underwent HRPK in the University Ophthalmic Hospital in Warsaw. Ninety-eight consecutive patients were treated with MMF and prospectively followed up. The control group included 98 consecutive patients assessed retrospectively. In 2009, results of a multicenter, prospective and randomized clinical study concerning the efficacy of mycophenolate mofetil after high-risk corneal transplantation were published [16]. Recruitment for the study’s control group was prematurely stopped due to a statistically significant difference between treated patients and the non-MMF-treated control group (in favor of the group treated with MMF). That is why the control group was assessed retrospectively in our study; we found it unethical not to administer effective therapy [16].

Informed consents were obtained, and the local bioethical committee approved the study. We defined high-risk keratoplasty as retransplanted keratoplasties, emergency transplantations in case of corneal perforation due to bacterial infections, presence of deep vascularization in three or four quadrants, and transplantation in patients with keratouveitis (Table 1). Exclusion criteria included perforation due to unknown cause, viral or fungal corneal infections, and glaucoma diagnosed prior to the surgery. The indications for corneal transplantation are listed in Table 2.

**Table 1:** Indications for HRPK (Fisher’s exact test: *p* = 0.247; no statistical difference)

Diagnosis	Group 1 (MMF)	Group 2 (control)	Total
3 or 4 quadrants with deep vascularization (*n*)	12	12	24
3 or 4 quadrants of retransplanted corneal button with deep vascularization	20	24	44
Emergency transplantations (*n*)	30	40	70
Retransplantations (*n*)	31	20	51
Active recurrent or chronic uveitis (*n*)	5	2	7

**Table 2:** Indication for the corneal transplant

Diagnosis	Group 1 (MMF) *n* = 98	Group 2 (control) *n* = 98	Total
Corneal opacity and neovascularization due to severe dry eye syndrome	1	2	3
Alkali burn	7	9	16
Trauma ulcer	4	1	5
Graft failure due to graft rejection	35	30	65
Graft failure due to nonhealing epithelial defect	4	3	11
Graft failure due to corneal melt	6	5	12
Graft failure due to bacterial corneal ulcer	6	6	14
Acute perforation of non-infectious corneal ulceration	3	4	7
Refractory bacterial keratitis	5	7	12
Acute perforation of corneal ulcer	22	29	51
Keratouveitis of idiopathic etiology	5	2	7

HLA and ABO blood group antigen matching had not been performed before the surgery.

All donor corneas were stored in Eusol GS medium at 4 °C (mean time of storage, 5 days). The mean endothelial cell density of donor corneal button was 2400 cells/mm^2^ (1700-3000 cells/mm^2^).

All patients underwent penetrating keratoplasty or penetrating keratoplasty with extracapsular cataract extraction (Table 3). The mean diameter of the donor corneal discs was 8.0 ± 0.5 mm, 0.5 mm larger than the diameter of the recipient bed. In cases of emergency keratoplasties, interrupted 10-0 nylon sutures were used; in other cases, a combination of interrupted and continuous 10-0 nylon sutures was applied. All knots were buried in the peripheral host cornea. Directly after surgery, all patients received antibiotic eye drops (fluoroquinolone), and subconjunctival injection of dexamethasone was administered.

**Table 3:** Type of surgery (χ^2^ = 1.42, *p* = 0,54; no statistical difference)

	Group 1 (MMF)	Group 2 (control)	Total
Penetrating keratoplasty (*n*)	68	72	140
Penetrating keratoplasty + extracapsular cataract extraction (*n*)	5	2	7
Penetrating keratoplasty + extracapsular cataract extraction + intraocular lens implantation (*n*)	25	24	49

Patient data are listed in Tables 3 and 4.

**Table 4:** Patient data

	Group 1 (MMF)	Group 2 (control)	Total	*p* value
Patients (*n*)	98	98	196	
Male/female (*n*)	51/47	42/56	93/103	0.253 (ns)^*^
Age (mean ± SD)	58 ± 16	56 ± 16		0.508 (ns)^**^
Follow-up (days)	394.9 ± 214	353.7 ± 270		0.238 (ns)^***^

ns, no statistical difference.

^*^Pearson’s χ^2^ test; ^**^Mann–Whitney *U* test; ^***^Student’s *t* test.

Group 1 was prospectively followed up and received mycophenolate mofetil (CellCept®, Roche Pharma AG, Grenzach-Wyhlen, Germany), according to a set protocol. All group 1 patients were examined by the transplantologist, and a thorough assessment was conducted comprising the patient’s history of peptic ulcer, malignancy, and chronic infections; blood pressure measurement; and laboratory tests, including complete blood cell count, serum creatinine, and liver functions. Afterwards, systemic immunosuppression was introduced. A day before surgery, MMF was administered at an initial dose of 2 × 1000 mg. On the day of surgery and for 2 days after, the patients received methylprednisolone sodium succinate intravenously (1st day, 500 mg; 2nd day, 250 mg; 3rd day, 250 mg). On the fourth day, methylprednisolone was administered orally (0.4 mg/kg daily). MPA serum concentration was measured on the seventh day after surgery (a predose MPA serum concentration reached about 2 μg/ml and no more than 5 μg/ml). Methylprednisolone was tapered during the first month after surgery to reach the lowest possible dose of 5–10 mg daily, and was discontinued after 11 months. A month after the operation, MMF was also tapered to 2 × 500 mg, after 6 months to 2 × 250 mg, and a year after surgery MMF was discontinued. Since immunosuppressive therapy may activate viral infections (e.g., herpes simplex virus, cytomegalovirus, human papillomavirus) and induce atypical infections (*Pneumocystis jirovecii*), prophylaxis for 6 months with acyclovir (4 × 400 mg daily) and co-trimazole (480 mg twice daily for 3 months tapered to 480 mg daily) was introduced. Apart from systemic treatment, topical steroid drops were administered (loteprednol etabonate).

Group 2 consisted of 98 consecutive patients assessed retrospectively with no systemic immunosuppressive therapy administered. On the day of the surgery and in the next 2 days after surgery, the patients received methylprednisolone sodium succinate intravenously (500 mg once daily). On the fourth day, the patients received methylprednisolone (1 mg/kg). The mean dose of methylprednisolone after a month was 0.5 mg/ kg daily; after 2 months, it was 0.3 mg/kg daily; and after 6 months, it was 5–10 mg daily.

Patients in both groups received (from the first day after surgery) topical antibiotic drops or ointment (fluoroquinolone) until epithelial healing was complete, and afterwards they received topical steroid for a year or longer, depending on their response, until all sutures were removed (loteprednol was administered in the MMF group and dexamethasone in the control groups four to six times daily for 2 months, three times daily until the sixth month, and two times daily thereafter for 6 months or longer).

Postoperative examinations were performed each day during the first week after surgery, and then on the following schedule: 1, 3, 6, 12, and 24 months after surgery. The examination included visual acuity for distance and near vision, intraocular pressure measurement, and slit lamp examination. Apart from the ophthalmic examination, each patient was examined by the transplantologist. Side effects (gastrointestinal, hematologic) and MPA plasma concentration were closely monitored. Predose MPA plasma concentration was measured a week after surgery, a month after surgery, 3 months after surgery, and each time the dosage of MMF was changed (during the follow-up time, an average of three measurements were taken).

The main outcomes of the study were occurrence of immune graft rejection and graft survival. Immune reactions were diagnosed on the basis of a patient’s complaints (redness, discomfort, pain, worse visual acuity, photophobia, lacrimation) and slit lamp examination findings, such as endothelial precipitates, Khodadoust line, noninfectious subepithelial infiltrates, and stromal edema [17]. In case of immune graft rejection, patients in both groups were treated with topical dexamethasone drops every hour and with ointment at bedtime. In case of severe reactions (more than five keratic precipitates, inflammatory cells in stroma not due to infection, endothelial rejection line, stromal oedema, cells in aqueous humor) [18], the patients (group 1 and 2) were admitted to hospital and treated with intravenous methylprednisolone sodium succinate (500 mg for 3 days) and oral methylprednisolone acetate (1 mg/kg) afterwards, in addition to topical treatment as mentioned above. Patients in group 1 also had their MMF dosage increased to 2 × 1000 mg daily.

Clear graft survival was determined when there was no opacity in the 4-mm central zone of the corneal button. Graft failure was diagnosed with the presence of irreversible corneal edema and graft opacity.

## Statistical analysis

Statistical analysis was carried out using R statistical software.

Groups 1 and 2 were compared using Student’s *t*-test (patient age, visual acuity, graft diameter, follow-up time) and Mann–Whitney *U* test (endothelial cell density, donor age, donor tissue storage time). Chi-squared test was performed to compare surgical procedures employed. The Kaplan–Meier estimator was used to establish clear graft survival and rejection-free interval. Statistical significance was determined using the log-rank test. For all tests, a *p* value below 0.05 was considered statistically significant. Proportional hazards model (Cox regression) was used to establish risk factors of graft rejection.

## Results

### Demography

One hundred and ninety-six consecutive patients (103 women and 93 men, aged 21–92 years) were enrolled in the study. Of these, 98 were prospectively followed up and treated with systemic immunosuppression with MMF (group 1), and 98 were included in a retrospective control group with no systemic immunosuppression with MMF administered (group 2). None of the patients were lost for the follow-up. Mean follow-up time was 56 ± 31 weeks in group 1 and 51 ± 39 weeks in group 2.

There was no statistically significant difference between the two groups regarding preoperative best correct visual acuity, recipient age, donor age, quality of the donor corneal disc, tissue storage time, preoperative graft endothelial cell density, graft diameter, or the type of surgery performed.

Patient data are shown in Tables 1, 2, 3 and 4.

### Efficacy

The mean time of observation was 95 weeks (94 ± 21 weeks in group 1 and 97 ± 18 weeks in group 2). At this time, 84 of all 196 patients (43 %) experienced immune graft rejection, and graft failure due to immune reactions occurred in 33 patients (almost 17 %). Graft failure due to other causes occurred in only 10 cases (5 %).

In group 1, immune reactions occurred in eight cases (8 %) during and despite MMF treatment, and five of them were reversible; in four cases (50 %), the rejection was treated as severe reaction. Graft failure due to graft rejection occurred in three cases (3 % of patients). Another six cases (6 %) experienced graft failures due to other causes (nonhealing, persistent erosions, glaucoma, or infection). Regarding the underlying diagnosis: 50 % of the rejected emergency and repeated transplants failed (lost transparency). In case of patients with retransplanted vascularized corneas, none of the rejected grafts lost transparency and none of the patients with the keratouveitis rejected the graft (Tables 5 and 6).

**Table 5:** Efficacy

	Group 1 (MMF)	Group 2 (control)	*p* value^*^
Graft rejection (*n*)	8	76	< 0.01
Graft failure due to graft rejection (*n*)	3	30	< 0.01
Graft failure due to other causes (*n*)	6	4	
Nonhealing, persistent epithelial defects (*n*)	4	3	
Glaucoma decompensation (*n*)	1	1	
Infection (*n*)	1	0	

^*^log rank test

**Table 6:** Rejected and failed grafts based on the underlying diagnosis

Diagnosis	Group 1 (MMF)		Group 2 (control)	
	Rejection (*n* = 8)	Failure (*n* = 3)	Rejection (*n* = 76)	Failure (*n* = 30)
3 or 4 quadrants with deep vascularization (*n = 12*)	0	0 (0 %)	8	0 (0 %)
3 or 4 quadrants of retransplanted corneal button with deep vascularization (*n = 20*)	2	0 (0 %)	18	8 (44 %)
Emergency transplantations (*n = 30*)	4	2 (50 %)	20	10 (50 %)
Retransplantations (*n = 31*)	2	1 (50 %)	29	12 (41 %)
Active recurrent or chronic uveitis (*n = 5*)	0	0 (0 %)	1	0 (0 %)

In group 2, graft rejection occurred in 76 cases (77 %); in 15 of these cases, there was more than one episode of immune reaction during the follow-up, and in 33 cases (43 %) the rejection was severe. In 45 of the 76 cases, the reaction was reversible. Failure due to graft rejection occurred in 30 cases, and other causes for graft failure (nonhealing, persistent erosions, glaucoma) occurred in four cases. Regarding the underlying diagnosis: 50 % of the rejected emergency grafts, 40 % of the repeated transplants and 44 % retransplants with vascularized corneal bed failed (lost transparency). None of the grafts of patients with uveitis and immune reaction after the transplantation failed (Tables 5 and 6).

Efficacy data are shown in Tables 5 and 6.

**Fig. 1:**
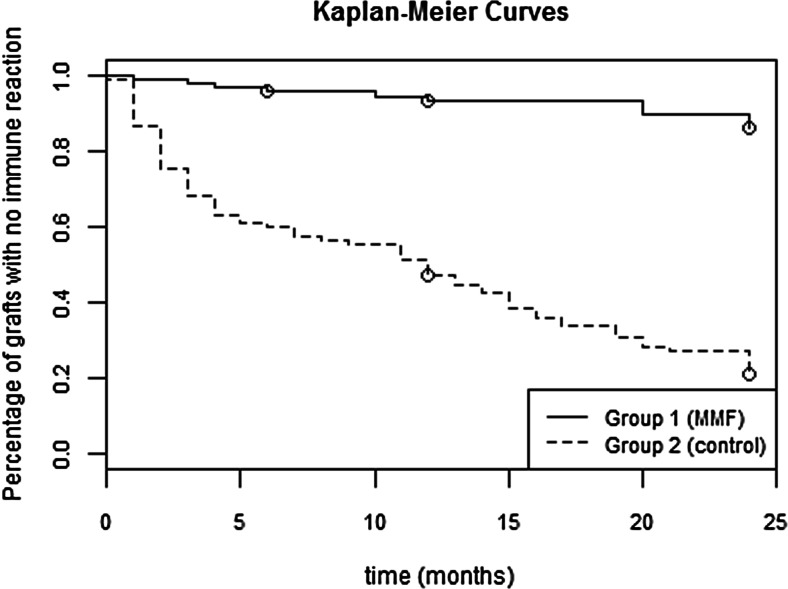
Kaplan-Meier survival curve concerning immune rejection free graft survival (log-rank test: *p* < 0.01)

According to Kaplan–Meier curves (Fig. 1), after 12 months of therapy, grafts without immune rejections accounted for 93 % of group 1 (MMF treated) and 47 % of group 2 (control group) (*p* < 0.01 in log-rank test). Cox regression analysis proved that MMF treatment decreased the risk of graft rejection 11 times (95.0 % CI 4.8–25, *p* < 0.0001). Furthermore, the other group characteristics (recipient’s age and sex, quality of the transplanted tissue, endothelial cell count of the donor tissue, diameter of the corneal button, donor age, time of storage, diagnosis, type of the surgery) included in the model had no effect on graft rejection. This indicates that the diminished risk of an immune response observed in group 1 (MMF treated) was a result of the therapy, and other factors that characterized the treatment group (recipient’s age and sex, quality of the transplanted tissue, endothelial cell count of the donor tissue, diameter of the corneal button, donor age, time of storage, diagnosis, type of the surgery) did not influence the result.

**Fig. 2:**
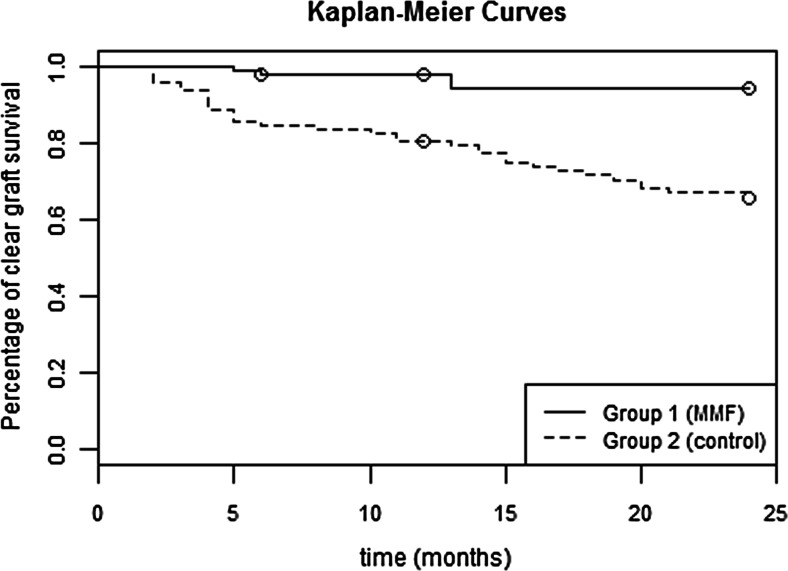
Kaplan-Meier survival curve concerning clear graft survival (log-rank test: *p* < 0.01)

The Kaplan–Meier plot illustrating clear graft survival (Fig. 2) shows that after a year of therapy, only 2 % of grafts failed in the MMF group compared to 19 % in the control group.

### Safety

In the MMF group, 13 patients (13.5 %) complained of gastrointestinal disturbances, but immunosuppressive treatment only had to be stopped in one case with refractive diarrhea. In other cases, proton pump inhibitors or MMF enteric-coated mycophenolate sodium were administered, and the discomfort subsided.

There were also two cases of anemia and three of leucopenia, which resolved after reduction of the MMF dosage.

## Discussion

There are three main immunologic risk factors for graft rejection: vascularization, inflammation, and previous graft rejection [19, 20], which we accounted for in the inclusion criteria of our study. In the Collaborative Corneal Transplantation Studies (CCTS) high-risk keratoplasty was defined as having two or more quadrants with deep stromal vessels; if corneas had vascularization in four quadrants, the risk for immune graft rejection doubled [4]. These findings were confirmed in other studies. The depth and extent of vessels seem to be critical factors for corneal graft survival (the more vessels, the higher risk for graft rejection) [4, 20, 21]. Active inflammation at the time of surgery is also a risk factor for corneal graft rejection and graft failure [4, 5, 7] as well as regrafting, especially if the failure was the result of immune graft rejection [4, 6, 21].

Topical application of corticosteroids is the gold standard after low-risk keratoplasties because it provides effective immunosuppression and good anterior chamber penetration [22]. But the impact of topical steroids is often insufficient in preventing graft rejection in high-risk patients. These procedures require an increased use of the eye drops, which can cause side effects such as cataract, glaucoma, or delayed wound healing. These impediments explain why the search for optimal treatment schedules for such patients remains ongoing.

To our knowledge, only two immunosuppressives are routinely used after high-risk keratoplasties: CsA and MMF.

CsA was introduced in ophthalmology in the mid-1980s, and its use in ophthalmology is well established. Many publications report good effects from CsA use after HRPK, but some report no benefits. In a meta-analysis of randomized controlled trials with two studies on CsA versus placebo, pooled analysis of clear graft survival showed no differences between the two groups [23]. CsA may be the cause of significant side effects, including hypertension, nephrotoxicity, neurotoxicity, and malignancies [10, 11, 24, 25].

In a prospective, randomized, multicenter study conducted by Birnbaum et al. [9], MMF was administered for 6 months after high-risk keratoplasty. In this study, HLA loci were typed, but the HLA matching was not performed. In the MMF group (*n* = 50), there were eight cases (16 %) of graft rejections, and two of them were irreversible; whereas, in the control group (*n* = 37), there were 12 cases (32 %) of immune graft rejection and seven were irreversible. The difference between the groups in graft rejection episodes was statistically significant (*p* = 0.044). It is worth mentioning that all rejection episodes in the MMF group occurred after cessation of MMF.

In our study, the patients were treated with MMF at full dosage (1000 mg twice daily) for about a month. Under optimal conditions, the dosage was tapered to 750 or 500 mg twice daily after a month and continued for the next 5 months. During the seventh month, the dosage was reduced to 2 x 250 mg daily, and MMF was discontinued after 12 months. In control group 2, the doses of methylprednisolone were higher than in the MMF group (1 mg/kg vs. 0.4 mg/kg at the beginning, 0.5 mg/kg vs. 5–10 mg daily after 2 months, 0.3 mg/kg vs. 5–10 mg daily after 3 months). The doses in both groups equaled after 6 months. It should also be stressed that most of the patients in group 2 (76 of 98, 77,6 %) did not follow the scheme, as when graft rejection occurred, the dosage of methylprednisolone was increased to 1 mg/kg daily, so mean doses of methylprednisolone were actually higher.

We also observed statistically significant differences in immune graft rejection and graft failure between the MMF and control groups (*p* < 0.01). There were eight episodes (8 %) of graft rejection in the MMF-treated group (*n* = 98), with three irreversible cases; in the control group (*n* = 98), there were 75 (almost 77 %) patients with graft rejection, and 30 of them eventually had graft failure. In the present study, about 40 % of grafts in both groups had irreversible immune rejection. Compared with the study of Birnbaum et al. [9], our groups were larger, which may explain why the effect of MMF treatment was even more pronounced.

Taking into account the underlying diagnosis, the same percentage of rejected emergency grafts in both groups failed (50 % of rejected MMF-treated and MMF-non-treated grafts lost transparency). These results are comparable to other studies, as other researchers found 25–62 % of emergency graft lost their transparency [26, 27].

As far as retransplantations are concerned, respectively, 50 % of grafts (one patient) in group 1 and 40 % of grafts (12 patients) in group 2 failed. The literature shows that about 33 to 46 % of repeated grafts fail in 2 years of follow-up [26, 28–30].

Forty-four percent of neovascularized regrafts failed in the control group, which is less than other researchers found (50-83 %) [26, 27].

The results are summarized in Tables 5 and 6. Emergency transplants and retransplantations seem to be high-risk factors for permanent loss of graft transparency. The main side effects in our MMF group (*n* = 98) were gastrointestinal disorders in 13 cases (13 %) and hematologic changes (anemia, leucopenia) in five cases (5 %). Apart from one patient with refractory diarrhea, all side effects were well tolerated and reversible. In the study of Birnbaum et al. [16], out of 57 MMF-treated patients, 36 (63 %) suffered from side effects, but the authors noted that they were probably the effect of systemic immunosuppression with MMF in only 24 cases (42 %). In that study, 15 patients (26 %) suffered from gastrointestinal disturbances; in one case, the MMF therapy was stopped, eight patients (14 %) developed infections, and one had anemia. The authors also reported two cases of malignancies (3 and 5 months after surgery) [16]. In the Reis et al. [12] study, Hodgkin’s lymphoma was diagnosed in the MMF group a month after surgery. Cancerogenesis due to MMF use cannot be excluded, but it seems unlikely. In 2005, a study based on data collected on almost 7000 patients in the Organ Procurement and Transplantation Network, United Network for Organ Sharing and Collaborative Transplant Study registry showed that there was no statistically significant difference between MMF-treated and non–MMF-treated de novo renal transplant recipients in 3 years of follow-up study for the development of lymphomas (*p* = 0.999) and other malignancies (*p* = 0.088) [15]. MMF is considered less active in cancerogenesis than CsA or tacrolimus, and treatment of less than 6 months seems too short to induce neoplastic disease. It is more probable that the patients were asymptomatic prior to keratoplasty. Nevertheless, other studies reported more side effects than we observed. Because MMF treatment seems rather safe, most authors do not monitor MPA serum level. Taking into account each patient’s variability and the dynamic relationship between the pharmacokinetics and pharmacodynamics of the drug, we believe that monitoring of MPA serum concentration should be mandatory, because it helps to avoid exceeding safe drug levels and permits individual dosage adjustment.

In conclusion, our study provides further evidence to confirm that systemic MMF treatment after HRPK considerably reduces the number of patients with immune graft rejection and graft failure. Most of the side effects are transient or reversible after adjusting the therapy, and the treatment is relatively safe. Since it was previously reported that pharmacologically induced immune tolerance may decrease after a certain time and MMF treatment may be insufficient, long-term graft survival studies need to be conducted [31].
